# Treatment of Appendicitis

**Published:** 1891-06-13

**Authors:** 


### SURGERY.

### TREATMENT OF APPENDICITIS.

Various nomenclatures have been adopted by different observers to indicate the part first affected in cases which come under the clinical head of "Typhlitis." But a consideration of the anatomy of the cæcum and its appendix will considerably modify and simplify some of these classifications. The blind pouch-like extremity of the cæcum—caput cæcum coli—is practically entirely covered by peritoneum, and so also is the appendix, which is attached by a thin mesentery to the wall of the rest of the cæcum. The appendix, moreover, very often hangs down into the pelvis between the cæcum and ileum, a point of some importance in the diagnosis of appendicitis. Dr. John Ferguson, of Toronto, has published the results of 200 dissections, which show that in 123 the appendix had a mesentery of its own, and in 77 was so placed and covered by peritoneum that a perforation might have opened into the subperitoneal tissue. These anatomical conditions do away with the idea of an inflammation starting in the supposed connective tissue around or behind the cæcum or appendix in the majority of cases, and enable us to divide them into those primarily affecting the cæcum, and those owing their origin to lesions in connection with the vermiform appendix.

As a broad general rule it may be stated that all severe cases of so-called typhlitis are really, in our proposed classification, cases of appendicitis—that is to say, have for their starting point some affection of the appendix.

The cases which we would include under the first head—true typhlitis—are usually caused by stercoral ulcers due to fæcal accumulations—possibly also by other irritating matter lodging in this part of the bowel—and they generally do well under the recognised medical treatment, and, except in very rare instances, never call for surgical interference.

Under our second head are included two varieties:—

I. Suppurative Typhlitis, as it is generally called, which generally owes its origin to perforation of the appendix. This may be brought about by ulceration, by twisting of the process inducing sloughing, by distension of the process by mucus—or, probably most often, by the impaction of a fæcal concretion or some foreign body, such as cherry or other fruit stones, pins, gall-stones, and the like.

II. Relapsing Typhlitis, or, more properly, Appendicitis. This variety differs from the last in that the trouble in the appendix falls short, at least for some time, of producing suppuration. The pathology of this affection is thus described by Treves: "The trouble is due to a retention of mucus within the vermiform process. The appendix is found twisted or bent upon itself, or held down at an angle by adhesions, or its lumen has been nearly occluded by cicatrisation after ulcer. The end of the process is found to be enormously distended with mucus, and often to be singularly hard. . . . There is no stone nor foreign body in these cases. The distended appendix sets up a certain amount of peritonitis, in time it relieves itself, and the patient enters a quiescent period, which may or may not precede another attack. In other and less frequent examples a foreign body has been found which had induced two, or possibly more, attacks before it caused suppuration."

These varieties often call for surgical interference sooner or later, and to them we will confine our remarks.

In every case of typhlitis the usual medical treatment will be first adopted, and early interference is much to be deprecated. Often the attack will subside, or, if going on to suppuration, this will be indicated by the usual signs, and is seldom pronounced before the fifth day. If suppuration occur, an encysted intra-peritoneal abscess is generally formed, the walls of the abscess being formed by agglutinated coils of intestine. The foreign body is expelled into the abscess, or the appendix sloughs, and with the evacuation of the pus the patient's troubles are at an end.

In such a case as this care should be taken to make the incision over the seat of suppuration where the pus can be let out directly without opening up the general peritoneal cavity, and the abscess walls should be interfered with as little as possible to avoid any risk of the same disaster. If the appendix is seen at once lying in the cavity it is quite legitimate and advisable to ligature the neck with catgut and remove the perforated portion, provided this is sufficiently far removed from the cæcum, otherwise it should be left.

Any fæcal fistula that remains will generally close up by gentle, regular washing out.

It is in the second variety—Relapsing Appendicitis—that surgical treatment is so markedly beneficial, and here the time and mode of operation is radically different.

With regard to the diagnosis of such cases, the history is, of course, of prime importance, and before surgical interference is justifiable this must be established; but, in addition, it is often possible to feel the distended process per rectum, owing to its anatomical position referred to above.

The presence of a tender spot at a point two inches from the anterior superior spine, on the line between it and the umbilicus, is drawn attention to by Dr. McBurney as a localising sign of high value, but is rather doubtful.

Radiating pains and disturbances of micturition are also likely to be present in many cases.

The relapses have a tendency to increase in severity, and hence it is of the utmost importance to operate as soon as the nature of the case is clear, as any unnecessary delay may give time for the development of a fresh attack, which may prove fatal; and as no treatment, however judicious, can guarantee any immunity, no delay can be recommended under this impression.

Operative treatment having been decided upon, a few precautions are necessary.

No operation must be undertaken before all inflammatory symptoms have subsided, but as soon after this as possible. It is recommended to open the abdominal wall just beyond the diseased area, so as to be free of adhesions. In this way the peritoneal cavity is more easily defined, and, moreover, the cæcum or appendix might be adherent to the abdominal wall, and so be injured. When these are exposed the rest of the cavity can be shut off by sponges. The appendix should be damped and cut off about half-an-inch from the cæcum.

Mr. Treves recommends uniting the mucous membrane edges first, and then the muscular walls, by numerous fine sutures; he says it is impossible to be sure of bringing the serous coats together. To still further secure against extravasation the stump might be lightly attached to any adjacent peritoneal surface.

During the operation any adhesions likely to give rise to trouble should be dealt with. No drainage tube is required, the abdominal wound being completely closed.

This operation when carefully carried out offers every prospect of a permanent cure, and ought to expose the patient to no undue risk.

